# Persistent Cholelithiasis Following Biochemical Resolution of Parenteral Nutrition-Associated Cholestasis in a Former Preterm Infant: Surgical Management With Open Cholecystectomy

**DOI:** 10.7759/cureus.116938

**Published:** 2026-09-25

**Authors:** Brayan A Flores-Zepeda, Cynthia P Celis-Recio, Jorge O Beltrán-Delgado, Diego A Sánchez-Flores, José E Soto-Valle

**Affiliations:** 1 General Surgery, Center for Research and Teaching in Health Sciences, Autonomous University of Sinaloa, Culiacan Civil Hospital, Sinaloa, MEX; 2 Pediatric Surgery, Sinaloa Pediatric Hospital Dr. Rigoberto Aguilar Pico, Sinaloa Health Services, Sinaloa, MEX

**Keywords:** biliary stasis, open surgical approach, pediatric cholelithiasis, pediatric hepatobiliary surgery, premature birth, total parenteral nutrition (tpn)

## Abstract

Gallstones in early infancy are increasingly detected in survivors of neonatal intensive care, and most contemporary series favor observation because spontaneous resolution is common. Less well described is the divergence that may follow: cholestasis resolves completely while the stones formed during it remain. We present the case of a male infant born at 35 weeks of gestation who required high-frequency oscillatory ventilation, five vasoactive agents for septic shock, and 23 days of parenteral nutrition during a 45-day admission, developing cholestatic liver disease (total bilirubin 11.9 mg/dL, alkaline phosphatase 1,657 U/L). Ultrasonography at 2.8 months showed a cluster of gallstones measuring 9.8 × 5 × 7.4 mm, without cholecystitis or duct dilation. The cholestasis then resolved completely, and growth was restored; at 7.3 months, total bilirubin was 0.15 mg/dL and gamma-glutamyl transferase was 8 U/L. The stones did not resolve, a 6.5 mm calculus persisted at 5.0 months, and the infant remained asymptomatic. After six months of observation, open cholecystectomy was performed at eight months and 26 days of age and 9.1 kg, without complication; a black pigment calculus was recovered, and histopathology showed changes of immaturity without inflammatory infiltrate. He was discharged on the second postoperative day and remained free of biliary symptoms through 102 days. Biochemical recovery of the liver does not predict clearance of established lithiasis, the lithogenic exposures in these infants are multiple and should not be attributed to parenteral nutrition alone, and operating on an asymptomatic infant deserves to be argued explicitly rather than assumed.

## Introduction

Cholelithiasis was once considered a curiosity in infancy. It is now recognized with increasing frequency, largely because survivors of neonatal intensive care are numerous and because abdominal ultrasonography is performed liberally in unwell infants for unrelated indications. Reported prevalence across the pediatric population is 1.9%-4%, and children under approximately two years of age account for a substantial minority of all pediatric cases [1,2].

Infants are not simply small children in this respect. The lithogenic exposures that dominate infancy - prematurity, prolonged fasting, sepsis, hemolysis, transfusion, diuretics, and parenteral nutrition - are largely absent in the adolescent population from which most pediatric surgical evidence is drawn [1,3]. Parenteral nutrition-associated cholestasis, increasingly subsumed within the broader concept of intestinal failure-associated liver disease, affects up to 85% of neonates receiving prolonged parenteral support, and its severity relates to the duration of parenteral nutrition, the absence of enteral feeding, recurrent sepsis, and the composition of the lipid emulsion [4,5].

The clinical difficulty is not diagnosis but disposition. Most infant gallstones are asymptomatic and many resolve spontaneously, which argues for observation; yet resolution is not universal, no consensus guideline addresses the asymptomatic infant, and the threshold at which continued observation becomes unjustifiable has never been defined [1,6]. Compounding this, hepatic recovery and biliary clearance are usually discussed as though they proceeded in parallel, and clinicians may reasonably but wrongly interpret normalizing liver biochemistry as evidence that the biliary problem is resolving.

We present an infant in whom parenteral nutrition-associated cholestasis resolved completely and nutritional status was fully restored, while the gallstones persisted unchanged through six months of documented observation before cholecystectomy was undertaken. Our aim is to use this dissociation to examine a question for which the evidence remains thin: in an asymptomatic infant whose liver has recovered, how long should gallstones be observed, and what should shift the balance toward operation?

## Case presentation

A male infant was delivered by cesarean section for breech presentation at 35 weeks of gestation (Capurro assessment), weighing 3,346 g. The pregnancy had been complicated by polyhydramnios and a third-trimester maternal urinary tract infection, and advanced resuscitation with tracheal intubation was required at birth.

The neonatal admission lasted 45 days. Respiratory distress syndrome was treated with two doses of surfactant, high-frequency oscillatory ventilation for six days, conventional ventilation for a further 14 days, and nasal continuous positive airway pressure for seven days. The course was complicated by severe infrasystemic persistent pulmonary hypertension with biventricular hypertrophy, requiring five vasoactive agents; by septic shock with *Staphylococcus haemolyticus* bacteremia, treated sequentially with ampicillin and amikacin, then cefepime, then meropenem and vancomycin for 16 days; by intracranial hemorrhage; and by metabolic acidosis. He received three transfusion episodes and parenteral nutrition for 23 days. Ceftriaxone was not administered at any time during the neonatal admission. He was discharged with cholestatic liver disease on propranolol, phenobarbital, and ursodeoxycholic acid. The complete clinical sequence is summarized in Table 1, and biochemistry at 43 days showed marked conjugated hyperbilirubinemia with a disproportionate rise in alkaline phosphatase (Table 2).

**Table 1:** Clinical timeline. ALP: alkaline phosphatase; ASA: American Society of Anesthesiologists physical status; AST: aspartate aminotransferase; HFOV: high-frequency oscillatory ventilation; mo: months; nCPAP: nasal continuous positive airway pressure; POD: postoperative day; PPHN: persistent pulmonary hypertension of the newborn Created by the authors.

Age (chronological)	Event
Birth	Cesarean delivery at 35 weeks of gestation for breech presentation; birth weight 3,346 g, length 49 cm; advanced resuscitation with intubation
0-45 days	Neonatal intensive care: surfactant ×2, HFOV 6 days, conventional ventilation 14 days, nCPAP 7 days; septic shock (*Staphylococcus haemolyticus*) with five vasoactive agents; severe PPHN with biventricular hypertrophy; intracranial hemorrhage; three transfusion episodes; parenteral nutrition 23 days; no ceftriaxone exposure
1.4-1.5 mo	Cholestatic liver disease; biochemistry at 43 days, discharge at 45 days: total bilirubin 11.9 mg/dL, direct bilirubin 7.0 mg/dL, ALP 1,657 U/L, AST 540 U/L. Ursodeoxycholic acid commenced
1.7 mo	First outpatient review: jaundice with bronzed appearance, hepatomegaly. Hepatobiliary ultrasound requested but not performed
2.7 mo	Readmission with acute bronchiolitis; nCPAP; vancomycin 11 days, cefepime 8 days, as-needed furosemide
2.8 mo	Cholelithiasis detected incidentally: conglomerate 9.8 × 5 × 7.4 mm plus 1.4 mm infundibular calculus; wall 1.9 mm; no cholecystitis; no ductal dilation. Surgery deferred
3.2 mo	Discharged after 15 days, without an outpatient appointment being issued
3.9 mo	Asymptomatic and anicteric, but severe wasting (weight-for-length z −3.2). Referred to pediatric surgery
4.4 mo	Cholestasis biochemically resolved: total bilirubin 0.53 mg/dL, direct bilirubin 0.33 mg/dL, albumin 4.27 g/dL
5.0 mo	Repeat ultrasound: calculus persists (~6.5 mm) in a gallbladder of 2.30 × 1.17 cm, wall 1.3 mm (Figure *1*)
5.0-8.1 mo	129 days without documented outpatient review
8.1 mo	Pediatric surgical review: asymptomatic; weight 9.125 kg, length 72 cm (normal growth restored). Cholecystectomy offered and accepted
8.8 mo	Open cholecystectomy at 9.1 kg; ASA II; single cystic duct; minimal blood loss; no intraoperative complication; pigment calculus recovered (Figures *2*, *3*)
8.8 mo, POD 1	Breastfeeding and puree diet resumed; subhepatic drain removed; nutrition assessment 9.5 kg, weight-for-length z +0.82 (normal weight)
8.8 mo, POD 2	Discharged home; total hospital stay 3 days; oral cefixime 5 days, paracetamol, lactulose
10.0 mo (POD 35)	Gastroenterology: 5 cm subcostal scar well approximated, no organomegaly, eutrophic (9.73 kg, P66). Histopathology recorded as pending; postoperative ultrasound requested
10.1 mo (POD 38)	Histopathology reported: gallbladder 4.1 × 3.0 cm; morphological changes of immaturity; no inflammatory infiltrate
10.2 mo (POD 41)	Surgical review: wound healed, no hyperemia or dehiscence; 9.53 kg, 73 cm
12.2 mo (POD 102)	Latest documented contact: no abdominal complaints; neurodevelopmental follow-up ongoing

**Table 2:** Serial liver biochemistry from the neonatal period to the preoperative assessment. ALP: alkaline phosphatase; ALT: alanine aminotransferase; AST: aspartate aminotransferase; GGT: gamma-glutamyl transferase; INR: international normalized ratio; mo: months Created by the authors from the institutional clinical record. Reference ranges are conventional pediatric values given for orientation; laboratory-specific ranges were not recorded in the source documentation. Dashes indicate that the parameter was not measured on that occasion. Cholelithiasis was first detected at 2.8 months of age, between the second and third columns; the final column is the last biochemistry obtained before cholecystectomy.

Parameter	43 days (1.4 mo)	2.9 mo	3.0 mo	4.4 mo	7.3 mo (preoperative)	Typical pediatric range
Total bilirubin (mg/dL)	11.9	-	-	0.53	0.15	0.2-1.2
Direct bilirubin (mg/dL)	7.0	-	-	0.33	0.08	<0.3
Indirect bilirubin (mg/dL)	4.88	-	-	0.20	0.07	<1.0
AST (U/L)	540	-	60	103	94	<40
ALT (U/L)	485	-	63	181	73	<40
ALP (U/L)	1,657	-	379	379	310	100-400 (infants)
GGT (U/L)	-	-	192	111	8	<50
Albumin (g/dL)	3.44	-	-	4.27	4.07	3.5-5.0
Prothrombin time (s)/INR	-	13.6/1.01	-	14.3/1.05	13.3/0.98	11-14/<1.2
Hemoglobin (g/dL)	12.4	-	10.3	11.6	-	10.5-13.5
Platelets (×10³/µL)	318	-	275	468	-	150-450

**Figure 1:**
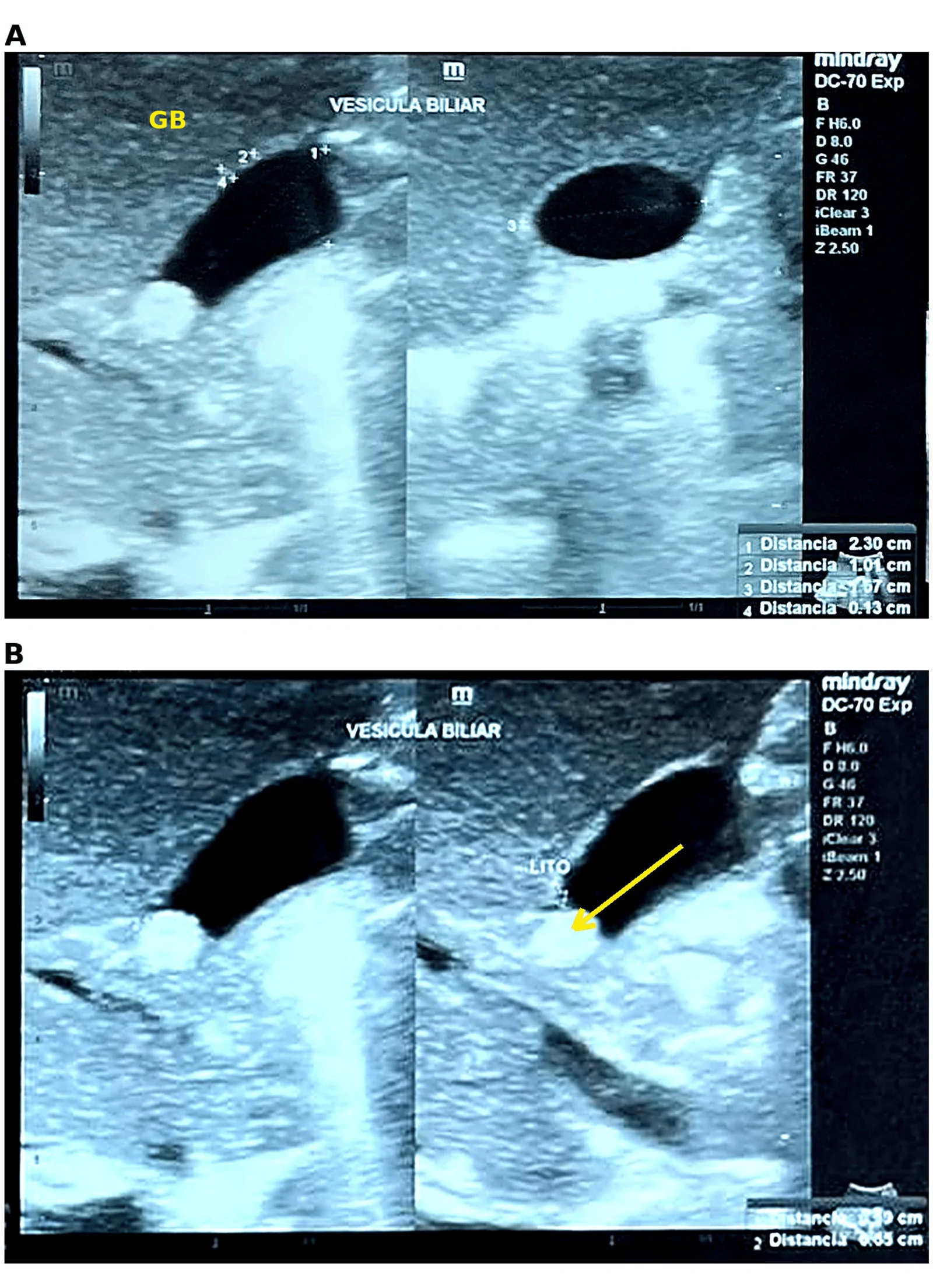
Abdominal ultrasonography at 5.0 months of age demonstrating persistence of cholelithiasis. (A) The gallbladder (GB) measured 2.30 cm in length and 1.17 cm in transverse diameter, with a wall thickness of 0.13 cm. (B) A single echogenic calculus of approximately 6.5 mm within the GB lumen (yellow arrow). The common bile duct was not dilated. On-screen annotations generated by the ultrasound system are in Spanish (“vesícula biliar”: gallbladder; “lito”: calculus; “distancia”: distance). Studies acquired on a Mindray DC-70 Exp system (Mindray Bio-Medical Electronics Co. Ltd., Shenzhen, China) with an SC6-1E curved-array transducer.

**Figure 2:**
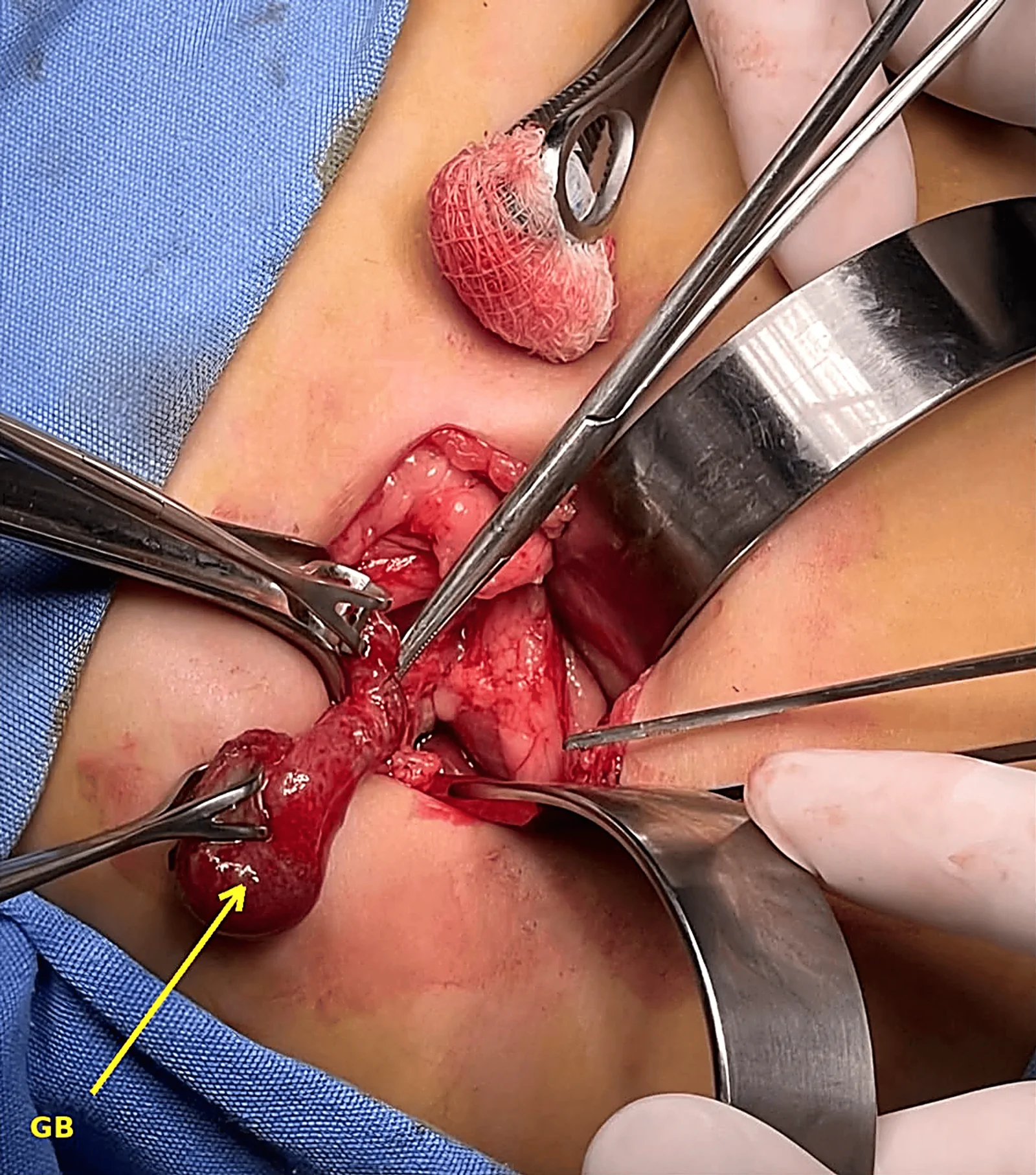
Intraoperative view during open cholecystectomy. The gallbladder (GB, yellow arrow) is retracted inferolaterally with Babcock forceps after dissection from the fundus toward the infundibulum. The hepatocystic triangle has been dissected, and the cystic artery is clamped prior to ligation and division; a single cystic duct was subsequently identified and confirmed before division. The 6 cm right subcostal incision and the scale of the operative field in an infant weighing 9.1 kg are apparent. Annotation added by the authors using Word (Microsoft® Corp., Redmond, WA, USA).

**Figure 3:**
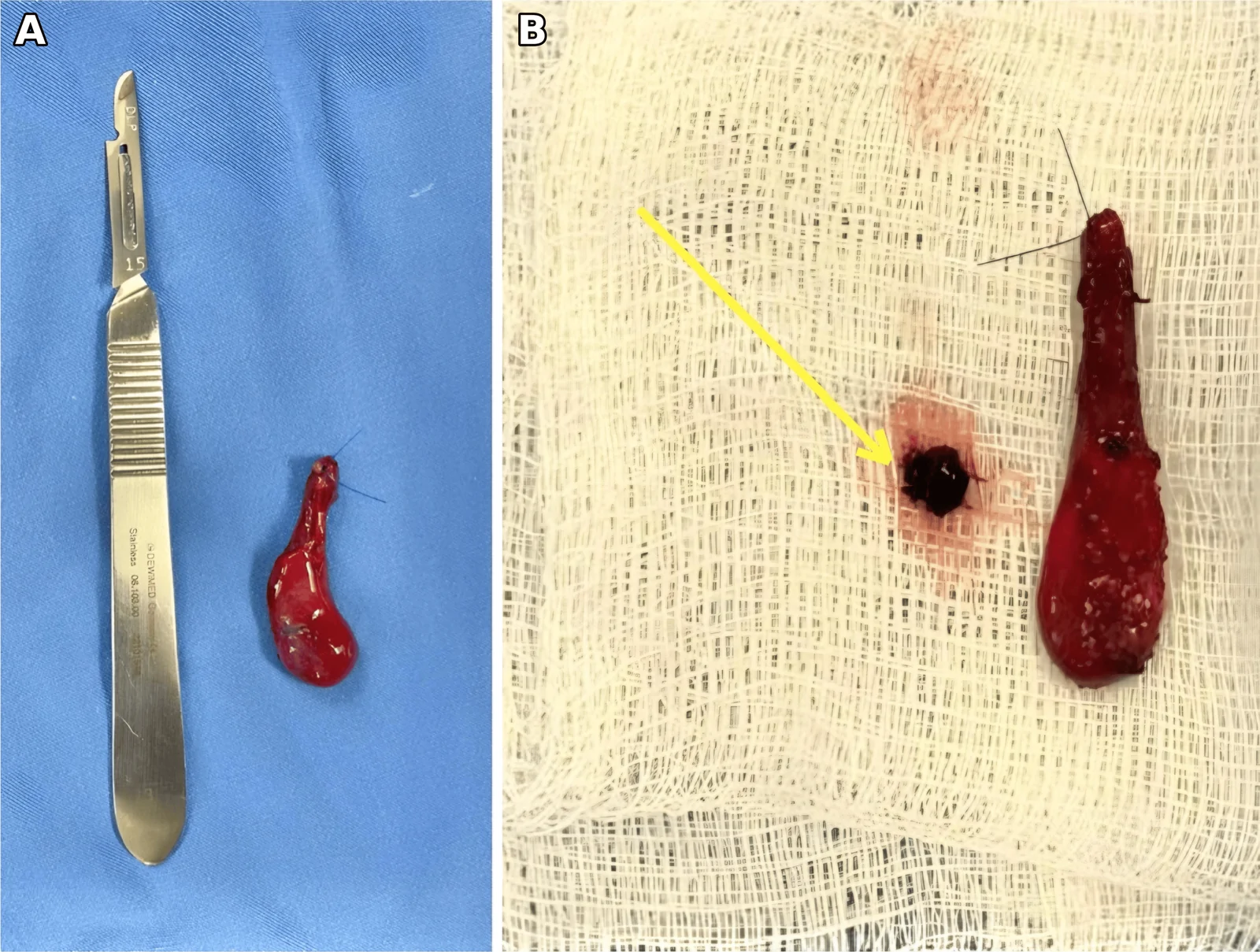
Cholecystectomy specimen. (A) The intact gallbladder alongside a scalpel bearing a No. 15 blade for scale, with the ligated cystic duct stump visible at the upper pole. (B) The opened specimen on a surgical swab with the recovered calculus (yellow arrow): a single black, friable stone whose macroscopic appearance is consistent with a pigment stone. Composition was not analyzed. Panel letters and arrow added by the authors using Word (Microsoft® Corp., Redmond, WA, USA). No anatomical feature of the images was altered.

At the first outpatient assessment (1.7 months), he was deeply jaundiced with a palpable liver; hepatobiliary ultrasonography was requested but not performed. At 2.7 months, he was readmitted with acute bronchiolitis requiring nasal continuous positive airway pressure and received vancomycin, cefepime, and as-needed furosemide. Ultrasonography performed on the second day of that admission to investigate the persisting cholestasis demonstrated a conglomerate of gallstones measuring 9.8 × 5 × 7.4 mm and a separate 1.4 mm calculus within the infundibulum, with a gallbladder wall of 1.9 mm, no features of acute cholecystitis, and no biliary dilation. Gastroenterology considered the finding a probable consequence of prolonged parenteral nutrition; surgery was deferred until his general condition allowed.

The cholestasis resolved completely over the following months (Table 2). Synthetic function was never impaired. By 4.4 months, total bilirubin was 0.53 mg/dL and jaundice, choluria, and acholic stools had resolved; by 7.3 months, six weeks before operation, total bilirubin was 0.15 mg/dL and gamma-glutamyl transferase was 8 U/L. Nutritional status followed the same trajectory: from severe wasting at 3.9 months (5.23 kg, weight-for-length z-score −3.2) to 9.125 kg and 72 cm at 8.1 months, appropriate for age.

The gallstones did not follow. Repeat ultrasonography at 5.0 months (Figure 1) showed a gallbladder measuring 2.30 × 1.17 cm with a wall of 1.3 mm containing a single echogenic calculus of approximately 6.5 mm; the common bile duct was not dilated. Gallbladder length was below the range reported in fasted infants under one year (mean 3.3 ± 0.8 cm; range 2.8-4.7 cm), whereas the transverse diameter was within the reported range (mean 0.9 ± 0.4 cm) [7]. He then had no documented outpatient review for 129 days.

At 8.1 months, he remained entirely asymptomatic. Given a stone burden that had neither cleared nor diminished across six months of observation, recovery of growth and anesthetic fitness, and the practical difficulty this family had already experienced in maintaining outpatient follow-up, cholecystectomy was offered and accepted by the mother after discussion. Open cholecystectomy was performed at a chronological age of eight months and 26 days (corrected age approximately 7.7 months) at a weight of 9.1 kg. Anesthetic risk was graded American Society of Anesthesiologists (ASA) physical status II. Through a 6 cm right subcostal Kocher incision, the gallbladder was identified containing calculi, grasped at the fundus and body with Babcock forceps, and dissected from the fundus toward the infundibulum. The cystic artery was ligated with 3-0 polyglactin and divided; the cystic duct was then ligated with 2-0 polypropylene, and a single duct was confirmed before division (Figure 2). The liver bed was inspected, and no bile leak was identified. A subhepatic Penrose drain was brought out through a flank counter-incision, and the wound was closed in layers. Blood loss was minimal, and no intraoperative incident occurred. A single black, friable pigment calculus was recovered from the specimen at the operating table (Figure 3), and the gallbladder was submitted separately for histopathological examination. The written pathology report described the wall microscopically as loose connective tissue with vascular congestion and numerous irregular lumina of variable caliber lined by simple columnar epithelium, and gave the diagnosis as gallbladder with morphological changes of immaturity (Figure 4).

**Figure 4:**
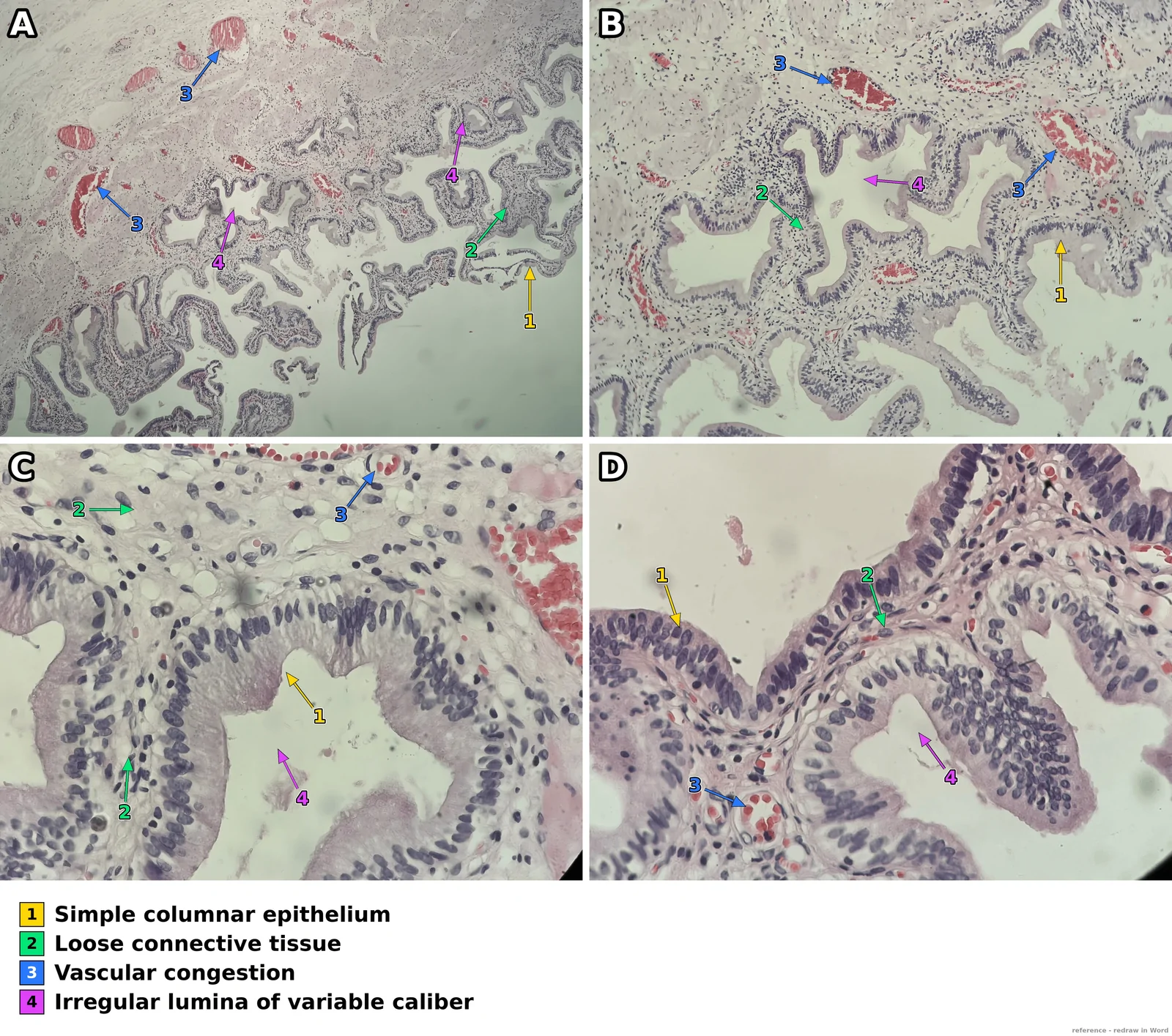
Hematoxylin and eosin (H&E) stain. (A) Irregular lumina of variable caliber and vascular congestion. (B) Lumina separated by loose connective tissue. (C) Simple columnar epithelium. (D) Adjacent lumina with congested capillaries. Numbered arrows are defined in the key. Annotation added by the authors using Word (Microsoft® Corp., Redmond, WA, USA).

The postoperative course was uneventful. Breastfeeding and a complementary puree diet were resumed on the first postoperative day, and the drain, which produced low-volume serous fluid, was removed the same day; formal anthropometry that day recorded 9.5 kg with a weight-for-length z-score of +0.82. He remained afebrile in room air and was discharged on the second postoperative day after a three-day stay.

Gastroenterology review 35 days after operation described a well-approximated 5 cm subcostal scar, no organomegaly, normal stool color, and eutrophic growth (9.73 kg, 66th percentile). Surgical review 41 days after operation found the wound healed without hyperemia or dehiscence. No biliary symptom, wound complication, or readmission for abdominal disease were documented at any point, and at the most recent contact 102 days after operation, he remained free of abdominal complaints.

Histopathology was reported 38 days after operation. Macroscopically, the gallbladder measured 4.1 × 3.0 cm, with a smooth green serosal surface and soft consistency. The written pathology report described the wall microscopically as loose connective tissue with vascular congestion and numerous irregular lumina of variable caliber lined by simple columnar epithelium, with no inflammatory infiltrate, and gave the diagnosis as gallbladder with morphological changes of immaturity.

## Discussion

Gallstones are reported in 1.9%-4% of children [2], but the age distribution is bimodal rather than linear: a first peak in infancy, driven almost entirely by perinatal and neonatal intensive care exposures, and a second in adolescence, driven by obesity and female sex [8]. Infants therefore behave as a distinct population, and evidence generated in adolescents transfers to them poorly.

The two largest contemporary pediatric surgical series illustrate the point. Among 147 children undergoing laparoscopic cholecystectomy, the median age was 16 years, with a single patient aged 15 months [8]; among 45 children, the mean age was 12.3 years, and no infant under one year was included [9]. A third series of 199 children found that symptomatic and complicated presentations clustered in those older than 10 years, and 96.5% underwent surgery [10]. Recommendations derived from such cohorts cannot be extended to an asymptomatic eight-month-old without acknowledging the extrapolation.

It would be convenient, and is frequently done, to attribute gallstones in an infant of this kind to parenteral nutrition. The record does not support so simple an account. Within a single 45-day admission, our patient accumulated four of the most common reported associations with infantile cholelithiasis - prematurity, sepsis, cephalosporin exposure, and parenteral nutrition, present respectively in 27.6%, 30.6%, 46.9%, and 29.6% of the 98 infants reported by Çakar et al. [1]. To these, he added a shock state requiring five vasoactive agents, three transfusion episodes delivering an increased bilirubin load, furosemide, and a period of enteral fasting whose duration is not recorded but which necessarily accompanied 23 days of parenteral nutrition. The association of parenteral nutrition with furosemide in premature infants who developed gallstones was described more than four decades ago, in precisely this combination [11]. Table 3 sets these exposures alongside their reported frequencies and proposed mechanisms, from sepsis-driven cholestasis within the spectrum of intestinal failure-associated liver disease [12] to the monogenic causes recently described in infants with biliary lithiasis [13].

**Table 3:** Risk factors associated with gallstone formation in neonates and infants, and their presence in this patient. FXR: farnesoid X receptor; HFOV: high-frequency oscillatory ventilation; IFALD: intestinal failure-associated liver disease Created by the authors. Frequencies are those reported by Çakar et al. in 98 infants aged 0-12 months with cholelithiasis [1] and are presented for context; they are not derived from this patient.

Risk factor	Proposed mechanism	Reported frequency in infants with cholelithiasis	Present in this patient
Prematurity	Immature bile acid transport and conjugation	27.6% [1]	Yes - 35 weeks
Sepsis	FXR downregulation; cholestasis of inflammation; ~3-fold increase in IFALD risk [12]	30.6% [1]	Yes - septic shock, *Staphylococcus haemolyticus*
Cephalosporin exposure	Biliary precipitation; ceftriaxone best characterized, causing reversible pseudolithiasis in 4.1%-27.3% of exposed children [2]	46.9% [1]	Yes - cefepime (two courses). No ceftriaxone before cholecystectomy
Parenteral nutrition	Cholestasis, phytosterol-mediated bile acid transport inhibition; risk rises beyond ~16.5 days in very preterm infants [5]	29.6% [1]	Yes - 23 days
Absence of enteral feeding	Loss of cholecystokinin-mediated gallbladder emptying, bile stasis, and concentration [4]	Not reported separately	Yes - duration not recorded
Hemolysis and transfusion	Increased unconjugated bilirubin available for calcium bilirubinate precipitation	Not reported separately	Yes - three transfusion episodes
Furosemide	Long-recognized association with gallstones in premature infants [11]	Not reported separately	Yes - as required
Congenital heart disease	Low-output states, hepatic congestion	18.4% [1]	Non-obstructive hypertrophic cardiomyopathy
Genetic disease	Includes trisomy 21; monogenic causes such as ABCB4 variants reported in infants [13]	16.3% [1]	Not investigated
Prolonged mechanical ventilation	Immobility and sedation, compounding fasting-related stasis	Not reported separately	Yes - 20 days invasive, 6 of them HFOV

One differential deserves explicit exclusion. Ceftriaxone-associated pseudolithiasis is the single most important mimic in this age group, occurs in 4.1%-27.3% of exposed children, and characteristically resolves spontaneously within 9-12 months of withdrawal [2]. Before cholecystectomy, our patient received ampicillin, amikacin, cefepime, meropenem, and vancomycin, but at no point received ceftriaxone; his only ceftriaxone exposure was perioperative, after the gallbladder had been removed. The persistence of his stones therefore cannot be attributed to a self-limiting drug precipitate.

The mechanisms operating in this population are additive rather than competing, and converge on three classical determinants: stasis, altered bile composition, and a nidus for precipitation. Stasis dominates. Absence of enteral nutrients removes the cholecystokinin-mediated stimulus to gallbladder contraction, so bile stagnates and becomes progressively concentrated; the joint European Society for Paediatric Gastroenterology, Hepatology and Nutrition/European Society for Clinical Nutrition and Metabolism/European Society of Paediatric Research/Chinese Society for Parenteral and Enteral Nutrition (ESPGHAN/ESPEN/ESPR/CSPEN) guidelines on pediatric parenteral nutrition identify absence of oral feeding among the disease-related factors impairing bile flow [4]. In our patient, the gallbladder measured 2.30 cm in length at 5.0 months, shorter than the reported range in fasted infants under one year (2.8-4.7 cm), while the transverse diameter of 1.17 cm was within the reported range [7]. However, the resected specimen measured 4.1 × 3.0 cm, indicating that the sonographic measurement reflected a contracted gallbladder rather than a structurally small organ. A single measurement cannot establish impaired contractility; the case for stasis therefore rests primarily on the documented absence of enteral feeding. Histology showed loose connective tissue with vascular congestion and numerous irregular epithelium-lined lumina, reported as morphological changes of immaturity, with no inflammatory infiltrate, consistent with the absence of cholecystitis throughout the clinical course, although the significance of these findings in a former preterm infant remains uncertain.

Bile composition is altered in several directions. Sepsis downregulates the farnesoid X receptor and increases the risk of intestinal failure-associated liver disease approximately threefold, while phytosterols in soy-based lipid emulsions antagonize bile acid transport and amplify hepatic macrophage inflammatory signaling [12]. Immaturity of hepatic bile acid transport and conjugation in the preterm infant adds a baseline vulnerability [2].

Finally, hemolysis and transfusion supply unconjugated bilirubin available for calcium bilirubinate precipitation. The black, friable calculus recovered at operation is morphologically consistent with a pigment stone, which is the expected phenotype when cholestasis, hemolysis, and biliary stasis coincide [2]. Composition was not analyzed, so this remains a morphological inference rather than a demonstrated fact.

Parenteral nutrition-associated cholestasis affects 40%-60% of pediatric patients and up to 85% of neonates receiving prolonged parenteral support, and gallbladder disease lies within the spectrum of intestinal failure-associated liver disease alongside cholestasis, steatohepatitis, and biliary cirrhosis [12]. Duration matters: in very preterm infants, parenteral nutrition beyond approximately 16.5 days is associated with a significant increase in cholestasis risk, while longer enteral feeding is protective in a dose-dependent fashion, with the parenteral-to-enteral ratio being the best single predictor [5]. Our patient received parenteral nutrition for 23 days, exceeding this threshold, and developed marked direct hyperbilirubinemia with preserved synthetic function.

What cannot be established from a single case is the step from cholestasis to lithiasis. Parenteral nutrition may have promoted stone formation through the cholestasis it caused, through the enteral fasting that accompanied it, or through both; concurrent sepsis, hemolysis, and diuretic exposure may also have contributed to biliary sludge. We therefore regard parenteral nutrition as one contributor among several and consider the lithiasis likely multifactorial. Parenteral nutrition is identified as a risk factor in 10%-17.6% of pediatric cholelithiasis cases overall [2]. The same exposures recur across the published experience: parenteral nutrition and furosemide in the two premature infants first described radiographically [11]; chronic hemolysis, glucose-6-phosphate dehydrogenase (G6PD) deficiency, and ceftriaxone in 35 Egyptian children, half of them under two years [3]; long-term parenteral nutrition among the contributors to a rising diagnosis in 199 children [10]; an incidental finding in an eight-week-old infant with neonatal jaundice [14]; and cephalosporins, sepsis, parenteral nutrition, and prematurity in 98 infants [1]. Table 4 sets these series alongside the present case.

**Table 4:** Comparative summary of published series and reports of cholelithiasis in infants and children associated with parenteral nutrition or critical illness. G6PD: glucose-6-phosphate dehydrogenase; UDCA: ursodeoxycholic acid Created by the authors from the cited sources. Entries are limited to reports in which parenteral nutrition, critical illness, or the neonatal period were explicitly described; this is a selective rather than a systematic compilation.

Study	Population	Exposures reported	Management	Outcome
Brill et al., 1982 [11]	Two premature infants with radiographically detected gallstones	Parenteral nutrition and furosemide; portal vein thrombosis in one	Not stated	Not stated
Enayet et al., 2020 [3]	35 children; 51.4% under two years	Chronic hemolysis 34.3%, G6PD deficiency 25.7%, ceftriaxone 17.1%, chronic cholestasis 8.6%	Conservative in 80%; laparoscopic cholecystectomy in 20%; UDCA in 12 symptomatic patients	Complete resolution 8.3% and partial response 33.3% with UDCA; choledocholithiasis in 20%
Wong et al., 2022 [10]	199 children, 2006-2020	Hemolytic disease 16.1%; long-term parenteral nutrition among the contributors to a rising diagnosis	Laparoscopic cholecystectomy in 96.5%	1.6% conversion; about 1% major postoperative complication; complicated disease in 14.5%
Brakohiapa et al., 2023 [14]	One infant aged 8 weeks, incidental detection	Neonatal jaundice with conjugated hyperbilirubinemia	Conservative	Reported as commonly self-limiting at this age
Çakar et al., 2024 [1]	98 infants aged 0-12 months; 87% asymptomatic	Cephalosporins 46.9%, sepsis 30.6%, parenteral nutrition 29.6%, prematurity 27.6%	UDCA in 90.8%; no cholecystectomy in any patient; four episodes of cholecystitis managed medically	Resolution 46.9%; 77.8% without UDCA versus 40.5% with regular UDCA (p = 0.03); poorer response in preterm infants
Singh et al., 2025 [13]	Two infants aged 9 and 2 months with choledocholithiasis	Neonatal jaundice; low birth weight in one; EHHADH and ABCB4 variants	Open cholecystectomy and Roux-en-Y hepaticojejunostomy after failed clearance	Clinical improvement with normalized liver function in both
Present case	One former preterm infant, asymptomatic throughout	Prematurity, septic shock, cefepime, parenteral nutrition for 23 days, enteral fasting, transfusion, furosemide	UDCA for about nine months; six months of documented observation; open cholecystectomy at 8.8 months	Cholestasis resolved while the stone persisted; uneventful course through 102 days of follow-up

The case for observing asymptomatic infant gallstones is genuinely strong, and any argument for operating must engage with it rather than ignore it. Among 98 infants aged 0-12 months, 87% were asymptomatic, gallstones resolved in 46.9%, four episodes of acute cholecystitis were all managed medically, no infant underwent cholecystectomy, and no endoscopic intervention was required [1]. In an Egyptian cohort of 35 children, of whom 51.4% were under two years, 80% were managed conservatively, and the authors concluded explicitly that conservative management is recommended for asymptomatic gallstones in children [3].

Three considerations qualify that position in a patient like ours. First, resolution is not universal: the 46.9% figure means a little over half of infants still had stones at the end of follow-up [1]. Second, size matters: only 2% of stones in that cohort exceeded 10 mm, whereas our patient’s initial conglomerate measured 9.8 × 5 × 7.4 mm, at the upper extreme of the distribution [1]. Third, and most relevant, resolution had already failed across six months during which every reversible contributor had been withdrawn, enteral nutrition was fully established, and liver biochemistry had returned entirely to normal. Observation in this case was not a hypothetical alternative never attempted; it was attempted, documented, and unsuccessful.

Ursodeoxycholic acid was continued in our patient for approximately nine months. Its value for the stones is a different matter, and here, the data are uncomfortable for common practice. In the infant series of Çakar et al., gallstones resolved in 77.8% of infants who received no ursodeoxycholic acid but in only 40.5% of those treated regularly for at least six months at 10 mg/kg/day (p = 0.03). The response was significantly poorer in preterm infants (p = 0.004), and the authors concluded that the drug should not be used routinely in infants outside symptomatic patients unfit for surgery [1]. That comparison is observational and almost certainly confounded by indication, since infants with larger or more complex stone burdens are precisely those likely to be treated, and it should not be read as evidence of harm. It does, however, argue strongly against expecting dissolution. Our patient was preterm and was treated for approximately nine months, and his stones did not dissolve - an outcome concordant with that literature.

No consensus guideline addresses the asymptomatic infant with persistent gallstones. Diez et al. reviewed 87 patients and concluded that dedicated guidance for asymptomatic pediatric gallstones is simply lacking, advocating early interdisciplinary consensus rather than fixed waiting periods [15]. Destro et al. offered the most usable formulation available: asymptomatic patients should be considered for surgery after a period of observation, taking account of age and pubertal maturation when other risk factors are absent [9]. Wong et al. likewise noted the absence of formal timing criteria while operating on 96.5% of their cohort [10]. Pelizzo et al., examining surgical timing directly, concluded that criteria used in adults cannot be transferred to children to gauge the severity of gallbladder damage, and called for pediatric-specific clinical and sonographic scoring [16]. None of these statements was derived from infants.

Where agreement does exist is at the complicated end of the spectrum. Symptomatic disease, acute cholecystitis, biliary pancreatitis, and choledocholithiasis warrant intervention. A stone impacted at the gallbladder neck and an underlying hemolytic disorder are reasonable additional indications, although neither has been validated in infants. Stone burden above 10 mm, failure to change across six months of structured observation, and unreliable access to follow-up remain matters of judgment for which no infant-specific evidence exists. Asymptomatic stones without any of these qualifiers are not a supported indication. The timing of surgery in an asymptomatic infant is the central and least resolved question, and we set out the reasoning applied in this case explicitly so that it can be disputed.

Four considerations argued for surgery. The stone burden lay at the upper extreme of the reported infant distribution. Resolution had failed completely across six months of documented observation, with all reversible contributors removed. Growth and anesthetic fitness had recovered to ASA II, whereas earlier operation would have been undertaken in a malnourished infant recovering from bronchiolitis. Finally, outpatient follow-up in this family had proved fragile: a 129-day interval elapsed without review, and an earlier discharge had occurred without any outpatient appointment being issued. That last factor is a health-system consideration rather than a physiological one, but where prompt re-presentation with acute cholecystitis or choledocholithiasis cannot be relied upon, we regard it as a legitimate element of the decision rather than an embarrassment to be omitted.

Arguing against surgery, and we state this plainly, were the facts that the infant was asymptomatic; that the largest contemporary infant series performed no cholecystectomies at all [1]; that complications of gallstones in this age group are infrequent; and that further observation would have been a defensible alternative. We do not claim that operating was the only reasonable course. We claim that it was a reasonable course, that the reasons were specific to this patient and his circumstances, and that such reasoning is more useful to readers when written down than when merely implied by the fact that an operation was performed. Figure 4 sets out the framework we would now apply, offered as a proposal derived from the literature reviewed rather than as a validated guideline.

**Figure 5:**
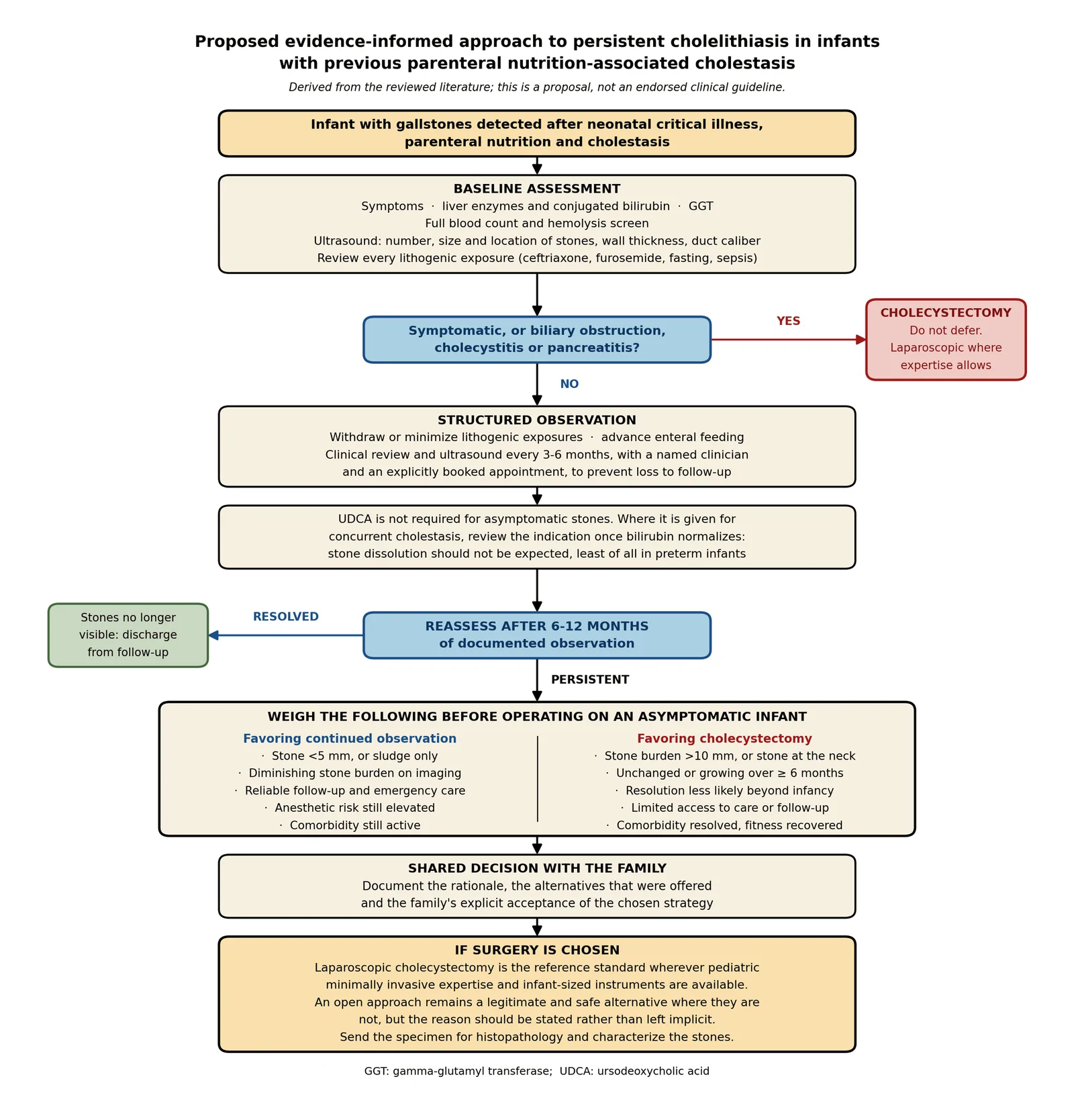
Proposed clinical approach to persistent cholelithiasis in infants with previous parenteral nutrition-associated cholestasis. The algorithm summarizes the proposed management pathway: exclusion of symptomatic or complicated disease, withdrawal of reversible lithogenic exposures with enteral feeding, structured observation, reassessment at 6-12 months, and explicit weighing of continued observation versus cholecystectomy before surgery in an asymptomatic infant. Amber indicates pathway entry/exit points, cream intermediate steps, blue decision nodes, red immediate cholecystectomy, and green discharge from follow-up. Created by the authors using Word (Microsoft® Corp., Redmond, WA, USA). This algorithm is a proposal derived from the literature reviewed in this report and has not been validated; it does not represent an endorsed clinical guideline.

Laparoscopic cholecystectomy is the reference standard for gallbladder disease in children, usually through a four-port technique and increasingly as day surgery [17]. Large pediatric series report conversion rates of 1.6%-2% and complication rates below 3% [8,10]. It has been performed in infants, and the technique is not intrinsically limited by age.

An open approach was chosen here on the basis of the instrumentation and pediatric minimally invasive expertise available at the time, and the procedure was completed through a 6 cm subcostal incision with identification of a single cystic duct, minimal blood loss, and no intraoperative complication. Open cholecystectomy remains in current use in the youngest patients, including in recently reported infants with complex biliary lithiasis [13].

We do not present the open approach as an advance. It is a pragmatic solution appropriate to a resource setting, and reporting it as such is more useful than presenting it as a technical contribution. The point worth retaining is that the choice of approach should be stated and justified in the operative record rather than left implicit.

Three clinical implications follow from this case. First, normalization of liver biochemistry is not a surrogate for clearance of stones. In this infant, bilirubin, alkaline phosphatase, and aminotransferases normalized, and nutritional status was fully restored while the stone burden did not change at all. Six weeks before operation, his liver biochemistry was entirely normal, with a total bilirubin of 0.15 mg/dL and a gamma-glutamyl transferase of 8 U/L; the calculus was nonetheless still present and was removed intact. Families and clinicians may reasonably read improving liver tests as evidence that the biliary problem is resolving; it is not, and the two require separate surveillance.

Second, ursodeoxycholic acid deserves a defined endpoint. Prescribed for cholestasis, it has a rationale; continued for months after cholestasis has resolved, in the expectation of dissolving established stones in a preterm infant, it is not supported by the available data [1].

Third, the failure that shaped this case most was one of continuity rather than of judgment. An earlier discharge without an outpatient appointment and a 129-day gap in review meant that observation, the strategy the literature favors, was never delivered as a structured program. Where surveillance cannot be guaranteed, the calculus that ordinarily favors watchful waiting shifts, and that is a health-system determinant of surgical decision-making.

This report has significant limitations, which we state fully. The calculus was recovered at the operating table and was not submitted with the specimen, so its composition was never analyzed, and its characterization as a pigment stone rests on macroscopic appearance alone. The microscopic findings are reproduced from the written institutional report and could not be illustrated, since no photomicrographs were obtained at the time of reporting and images of the histological sections could not subsequently be retrieved, in contrast to published pediatric cholecystectomy series in which histopathological findings are systematically catalogued [18]; the reported diagnosis of morphological changes of immaturity is descriptive rather than diagnostic, its prevalence in age-matched gallbladders is unknown, and we make no claim that it contributed to lithogenesis. A postoperative ultrasound was requested but not documented, so sonographic confirmation of a clear biliary tree after cholecystectomy is lacking.

Follow-up is documented to 102 days after operation, which is sufficient to exclude early surgical complications but too short to address the questions that matter most in this population: whether biliary symptoms, retained ductal stones, or recurrent lithiasis in the biliary remnant emerge over years rather than months. Under the CARE (CAse REport) reporting framework [19], the follow-up and outcomes domain is satisfied for the perioperative period but not for the longer term.

Genetic causes of infantile cholelithiasis were not investigated. Variants in ABCB4, which underlie low-phospholipid-associated cholelithiasis [2], and in genes affecting peroxisomal beta-oxidation have been identified in infants with biliary lithiasis, sometimes only after surgery [13]; a monogenic contribution in this patient cannot be excluded.

The composition and daily dosing of parenteral nutrition, including the lipid emulsion used, and the exact duration of complete enteral fasting were not recorded, which weakens any attempt to relate the lithiasis quantitatively to nutritional exposure. The exact start and stop dates of ursodeoxycholic acid are likewise not documented. Finally, the clinical record contains internal inconsistencies in several perinatal variables, including gestational age, birth weight, the duration of invasive ventilation, and the characterization of the intracranial hemorrhage. Where sources conflicted, we report the value from the note documenting an assessment method or closest in time to the event, and we have avoided reporting contested figures.

## Conclusions

Gallstones detected in an infant after neonatal critical illness are the product of several concurrent lithogenic exposures - prematurity, fasting, sepsis, hemolysis, transfusion, diuretics, and parenteral nutrition - and attributing them to parenteral nutrition alone overstates what a single case can support. In this infant, the hepatic consequences of that period reversed completely, with normalization of bilirubin, alkaline phosphatase, and aminotransferases, and with full nutritional recovery, while the gallstones persisted unchanged for six months. Biochemical resolution of cholestasis is therefore not a surrogate for resolution of lithiasis, and the two warrant separate surveillance. Cholecystectomy itself was uncomplicated, with enteral feeding resumed on the first postoperative day, discharge on the second, and no biliary or wound complication through 102 days of follow-up.

Most asymptomatic gallstones in infants can and should be observed, and ursodeoxycholic acid should be prescribed for cholestasis rather than in the expectation of dissolving stones, an expectation that the available infant data do not support and that is least likely to be met in preterm infants. Where observation has been given a fair trial and has failed, where the stone burden is substantial, and where growth and anesthetic fitness have recovered, cholecystectomy is a defensible option even in an asymptomatic infant, provided the reasoning - including any health-system considerations that influenced it - is set out explicitly and the family shares in the decision. A laparoscopic approach remains the reference standard; an open approach is an acceptable alternative where pediatric minimally invasive resources are limited, and saying so openly is more useful to readers than leaving it unexplained. What remains uncertain is the most important thing of all: how long a persistent stone in an asymptomatic infant may safely be watched, and that question will not be answered by case reports.
